# An Accessible Communication System for Population-Based Genetic Testing: Development and Usability Study

**DOI:** 10.2196/34055

**Published:** 2022-10-17

**Authors:** Tara Coffin, Deborah Bowen, Elizabeth Swisher, Karen Lu, Nadine Rayes, Barbara Norquist, Stephanie Blank, Douglas Levine, Jamie Bakkum-Gamez, Gini Fleming, Olufunmilayo Olopade, Alan D’Andrea, Denise Nebgen, Christine Peterson, Mark Munsell, Kathleen Gavin, Rebecca Lechner, Jamie Crase, Deborah Polinsky, Iris Romero

**Affiliations:** 1 University of Washington Seattle, WA United States; 2 MD Anderson Cancer Center Houston, TX United States; 3 Mount Sinai Medical Center New York, NY United States; 4 NYU Langone Health New York, NY United States; 5 Mayo Rochester, MN United States; 6 University of Chicago Chicago, IL United States; 7 Harvard Cambridge, MA United States; 8 Minnesota Ovarian Cancer Alliance Minneapolis, MN United States; 9 SHARE New York City, NY United States

**Keywords:** genetic testing, internet, social media, accessibility

## Abstract

**Background:**

Genetic testing uptake is low, despite the well-established connection between pathogenic variants in certain cancer-linked susceptibility genes and ovarian cancer risk. Given that most major insurers cover genetic testing for those with a family history suggestive of hereditary cancer, the issue may lie in access to genetic testing. Remotely accessible web-based communication systems may improve awareness, and uptake, of genetic testing services.

**Objective:**

This study aims to present the development and formative evaluation of the multistep web-based communication system required to support the implementation of, and access to, genetic testing.

**Methods:**

While designing the multistep web-based communication system, we considered various barriers and facilitators to genetic testing, guided by dimensions of accessibility. In addition to conducting usability testing, we performed ongoing assessments focusing on the function of the web-based system and participant response rates, with the goal of continuing to make modifications to the web-based communication system as it is in use.

**Results:**

The combined approach of usability testing and expert user experience consultation resulted in several modifications to the multistep web-based communication system, including changes that related to imagery and content, web accessibility, and general organization of the web-based system. All recommendations were made with the goal of improving the overall accessibility of the web-based communication system.

**Conclusions:**

A multistep web-based communication system appears to be an effective way to address many potential barriers to access, which may otherwise make genetic testing difficult for at-risk individuals to participate in. Importantly, some dimensions of access were easy to assess before study recruitment, but other aspects of the communication system required ongoing assessment during the implementation process of the Making Genetic Testing Accessible study.

## Introduction

### Background

The association between pathogenic variants in certain cancer-linked susceptibility genes, such as *BRCA1* and *BRCA2*, and an increased risk of ovarian cancer is well established [1-3]. Women with a personal or family history of breast or epithelial ovarian cancer are encouraged to undergo genetic counseling [4]. Current recommendations state that genetic testing leaves at-risk women better prepared to make decisions about cancer prevention, early detection, and treatment [5].

Despite these recommendations, only 15% to 30% of eligible patients, women with a personal history of ovarian cancer, are offered these services in clinical settings [4,6-10], and <20% of women with a first-degree relative diagnosed with breast or ovarian cancer are ever offered genetic testing [11,12]. As a result, it is estimated that <5% and 10% of women at elevated or high risk, respectively, ever receive appropriate genetic testing services [12]. Importantly, although nonclinical direct-to-consumer genetic testing, such as 23andMe and Ancestry DNA, is widely accessible, this service does not take the place of clinical grade genetic testing, which is still a required step in the verification process of commercial DNA test findings. As most major insurers offer coverage for testing for individuals who meet the US Preventative Services Task Force guidelines for genetic testing, including those with a personal or family history suggestive of hereditary cancer, the obstacle may lie with another facet of testing accessibility.

Health care access is a broad and multidimensional concept: these dimensions include approachability, acceptability, availability, affordability, and appropriateness [13]. Each dimension is critical in creating a truly accessible health resource. According to this model of accessibility, for genetic testing to be approachable, the service must exist, be reachable, and impact health outcomes [13]. Furthermore, the acceptability of current methods of genetic counseling and testing will impact the overall likelihood of testing [13]. Availability is dependent on the individual physically reaching genetic testing services in a timely manner [13]. Genetic testing must be universally affordable to everyone who may benefit from it, in that those who can potentially benefit from testing must be able to pay for it without a catastrophic expenditure of resources, including time and money. Finally, for genetic testing services to be appropriate, the services should meet the needs of the intended community, be perceived as appropriate by that population, and be provided at a time they can access it.

### Objective

As accessibility to genetic testing for at-risk individuals continues to lag, public health professionals face an urgent need to explore alternative approaches to genomic service implementation [14,15]. With this urgency in mind, we designed the Making Genetic Testing Accessible (MAGENTA) study to evaluate a new model of providing genetic testing that will potentially address the low uptake of genetic testing for women at risk for ovarian cancer. The MAGENTA study was a 4-arm noninferiority trial, using a multistep web-based communication system to deliver a combination of pre- and posttest electronic and phone-based genetic education and counseling and genetic testing results. Through this system, the MAGENTA study sought to better understand how to make genetic testing more accessible, increasing women’s ability to obtain genetic testing for ovarian cancer risk assessment. By providing genetic testing information and services on the web, individuals who can benefit from these services are arguably more likely to speak with their physicians or reach out to service providers and improve subsequent testing uptake.

The study team opted to conduct usability testing of the web-based communication system before opening study recruitment. The usability testing of MAGENTA’s web-based communication system was required to ensure that the system functioned as it was intended to, to identify bottlenecks in the enrollment process and to address general accessibility of the system itself, ensuring that participants could physically access information about the study. The MAGENTA study laid out a schema for increasing awareness of genetic education and counseling, potentially improving access by optimizing the usability of the web-based communication system before implementation. Here, we present the development and formative evaluation of the multistep web-based communication system required to support the implementation of genetic testing through web-based platforms that can be accessed anywhere there is an internet connection.

## Methods

### Overview

**Figure 1:**
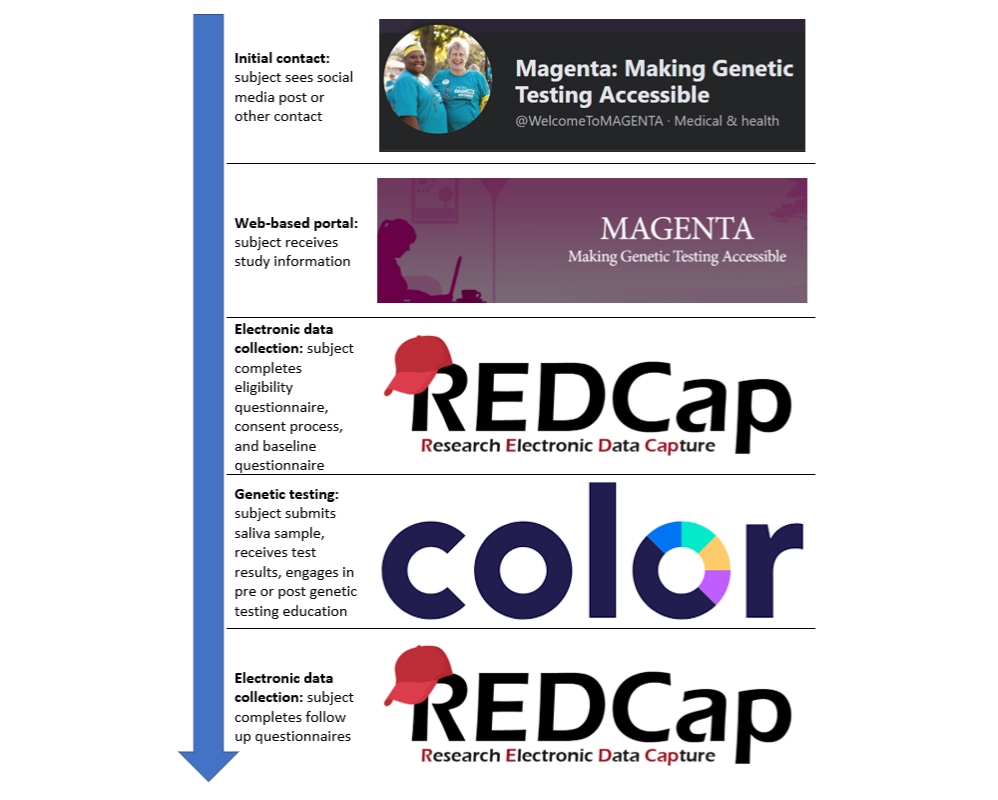
Overview of the web-based communication system for Making Genetic Testing Accessible (MAGENTA). This provides an overview of the flow for the web-based communication system developed and evaluated by the MAGENTA study team, with accessibility in mind.

The MAGENTA multistep web-based communication system sought to provide background information about the study, determine participant eligibility, guide participants through a web-based consent process, collect baseline data, walk participants through the web-based genetic testing protocol, and collect follow-up data. Figure 1 presents a flowchart of these components, emphasizing accessibility at each stage. Participants learned about the study on the web, in a clinical setting, or via a media outlet (eg, news article or story, and radio station). From there, they visited the MD Anderson MAGENTA website to review study information, learn more about eligibility criteria, and proceed to the eligibility questionnaire via a link to an electronic data collection tool. Eligible participants completed the informed consent process over an electronic data collection tool and received a follow-up email sending them back to the electronic data collection tool to complete baseline questionnaires. Once complete, participants were referred to Color Genomics, via a link over the email, where they begin the process of genetic testing. Participants receive their testing kit in the mail, engage in web-based genetic education with or without pre- and posttest genetic counseling over the phone, and receive their results on the web. At 3, 12, and 24 months following genetic testing, participants are prompted to return to the electronic data collection tool to complete follow-up questionnaires [16].

### Usability Testing

When using a web-based communication system that required many moving parts, it was critical to evaluate each component individually and as a system with the dimensions of accessibility in mind. In addition to an expert user experience (UX) consultation and ongoing collaboration with ovarian cancer advocates and clinicians, we conducted extensive usability testing. Usability testing, the process of evaluating the UX of interacting with a computer system, was implemented to assess the multistep web-based communication system such as this [17].

We recruited 10 cisgender women aged between 30 and 55 years, who had access to a computer, spoke English, and were located within driving distance of the University of Washington, to participate in usability testing. The eligibility criteria for usability testing mirrored the inclusion criteria for the MAGENTA study with the exception of geographic location; however, a personal or family history of cancer was not required. Most usability testing participants self-identified as non-Hispanic White. Participants responded to a flyer about the research and contacted the research team if they were interested in participating. If eligible, they were scheduled for a one-time in-person visit. At the start of this visit, participants signed a consent form and were informed about the usability study. Web-based components, including the initial study webpage and varying REDCap (Research Electronic Data Capture; Vanderbilt University) pages involved with the web-based communication system, were reviewed individually and when interacting with one another, capturing transitions between steps in the web-based communication system (Figure 1). Color Genomics was excluded from usability testing and expert UX consultation based out of the University of Washington. The Color Genomics system was not available for modification, so this portion of the web-based communication system was not assessed during usability testing. Color Genomics has extensive experience providing direct-to-consumer genetic testing services on the web and has previously undergone rigorous usability testing to assess its web-based platforms.

During testing, participants were prompted to “try to enroll in the study” or to “show me how you would go about learning more.” Components of the web-based communication system were reviewed in different formats, including how they would be viewed from a handheld mobile device or a computer, to assess the usability experience from different platforms. As participants responded to each component, they were encouraged to use the think-aloud approach, talking out loud about their expectations for how the web-based artifact functioned, prompted with follow-up questions encouraging them to speak more to their actions or thoughts concerning the system [17-20]. All participant-computer interactions were recorded using a screen-capture tool. At the end of the session, participants completed an adapted Post-Study System Usability Questionnaire developed by Lewis [21], which uses a 7-point Likert scale, where 1 indicates low satisfaction and 7 indicates high satisfaction, to measure user satisfaction with materials undergoing usability testing. The Post-Study System Usability Questionnaire developed by Lewis [21] examines system ease of use and efficiency, information quality, and quality of the system interface, in addition to the overall perception of the system. Participants who completed the questionnaire once were not asked to complete the questionnaire in subsequent usability testing. A total of 7 participants completed the poststudy usability questionnaire.

The resulting video data were coded in Atlas.ti (ATLAS.ti Scientific Software Development GmbH), a program well suited to code visual data. Data were coded using a content analysis approach. We used a deductive approach to coding, drawing on the dimensions of accessibility conceptualized by Levesque et al [13] to inform codebook development and analysis. The MAGENTA study team used the cumulative results from all evaluations to address usability issues, anticipate potential bottlenecks, and improve system accessibility. In the following section, we will review the process of designing and evaluating each of the components of the MAGENTA web-based communication system, a process guided by the dimensions of accessibility by Levesque et al [13].

### Dimensions of Accessibility

#### Overview

Access to health care is often reduced to a focus on financial and geographic barriers, hurdles that can be resolved with insurance coverage and reimbursement for transportation. These are not the only dimensions of accessibility that impact the uptake and use of health care resources, as illustrated by Levesque et al [13]. In addition to the availability of the services, which encompasses physical proximity of services, and affordability (cost), Levesque et al [13] described the approachability of a health care service, the acceptability of this service, and the appropriateness of the health care service in question, dimensions that are entangled with cultural competency. The concept of approachability is closely tied to awareness and health communication. An approachable health care service is one that a patient knows exists and understands how this service may impact their health and well-being [13]. Acceptability focuses on social factors that may influence uptake, including an individual’s comfort with a specific health care provider [13]. Finally, appropriateness speaks to the patients’ actual need, versus the need the service addresses [13]. We have discussed how each of these dimensions was evaluated during the development of the web-based communication system and through subsequent usability testing. In addition, Table 1 provides an overview of how the dimensions of accessibility were evaluated through each step of the assessment process.

**Table 1:** Summary of dimensions of accessibility and what methods were used to evaluate them.

Method and dimension		Assessment
**Usability testing and patient advocate review**		
	Approachability	Usability test participants and patient advocates provided feedback on the approachability of unpaid social media posts and paid Facebook advertisement, the MAGENTA^a^ study homepage, the REDCap^b^ study questionnaires and consent process, and all scheduled MAGENTA email communications
	Acceptability	Usability test participants and patient advocates provided feedback on the acceptability of images and text implemented across all written study components; familiarity of the components of the study, including the name of organizations associated with the research; and the REDCap study system, including consent process
	Availability	Usability test participants and patient advocates provided feedback on the availability of the general functionality of each component of the web-based communication system, including buttons and links; and the ability to locate study communications, including email
	Affordability	Usability test participants provided feedback on content related to cost of services
	Appropriateness	Usability test participants and patient advocates provided feedback on the appropriateness of images and text implemented across all written study components
Post-Study System Usability Questionnaire	Availability	Usability test participants completed the Post-Study System Usability Questionnaire developed by Lewis [21], after completing usability testing, providing general feedback concerning the usability and functionality of the web-based communication system
**Expert UX^c^**		
	Approachability	UX experts assessed the web-based communication system, focusing on web accessibility
	Availability	The UX consultation provided feedback on high-level workflows, page layout, and the usability of each web-based component
Ongoing system assessment	Availability	The study team tracked web analytics on an ongoing basis, across each web-based component in hopes of identifying and addressing any issues or bottlenecks before they impacted availability

^a^MAGENTA: Making Genetic Testing Accessible.

^b^REDCap: Research Electronic Data Capture.

^c^UX: user experience.

#### Approachability

We provided multiple points of entry, over multiple platforms, to make information about the study widely approachable. We generated a media kit that included internet-based outreach materials, including unpaid social media posts, paid Facebook advertisements, and other traditional outreach tools (eg, press release, flyers, and news articles). We opted to focus our web-based promotion efforts on Facebook because of its popularity as the most widely used web-based social media platform at the time of study recruitment [22]. Paid Facebook advertisements and unpaid social media posts included information about the study, a rotation of relevant imagery, and a link to the MAGENTA website. Unpaid social media posts included material shaped for use over Facebook, Twitter, YouTube, Instagram, and web-based blog sites.

In designing paid social media advertisements and unpaid social media posts, we drew on content and tone that would be more approachable to potential participants. Current research speaks to different approaches motivating people in varying ways [23]. For example, some may be more motivated to engage in a health behavior if they are doing it for their family, whereas others may have more self-serving interests [23]. Similarly, content that paints an image of teamwork and collaboration may facilitate engagement for some, but others may be more interested in acting independently [23]. We built posts and advertisements with this research in mind, in hopes of having a selection of materials that would resonate with different groups.

Unpaid posts were published to existing social media pages and groups by page administrators, on behalf of MAGENTA. These groups included Facebook pages run by breast and ovarian cancer advocacy organizations, gynecological cancer professional groups, and genetic testing companies. These groups were identified through keyword searchers and through referrals from patient advocates. By focusing on organizations with a vested interest in ovarian cancer genetics and an established community of followers, MAGENTA investigators aimed to leverage existing social networks, using familiar and trusted names to make information about the study more approachable [24].

Paid advertisements were posted to Facebook. All Facebook advertising campaigns targeted English-speaking women aged >30 years who live in the United States. After an initial soft launch period, we published a series of unpaid Facebook posts before moving to paid Facebook advertisements. We also used other traditional outreach efforts, including emails to clinicians and flyers in clinical settings.

It was critical that additional information was easy to locate and review. Knowing that participants may have found the study via several routes, we built a study website to introduce the multistep enrollment and participation process. All outreach efforts directed people to this website for study information and details about eligibility criteria. The MAGENTA study website also referred participants to the next step in the communication process via a link to the electronic data collection system.

We ran social media materials, the MAGENTA website, and each component of the electronic data collection system through usability testing to evaluate approachability. Usability testing focuses on whether a system is aesthetically and functionally accessible to the end user*,* evaluating if users could approach study materials as intended. We introduced participants to the study through Facebook posts and advertisements, prompting them to explain what they would do to learn more about the study. Participants also reviewed the MAGENTA website and the electronic data collection system in this fashion. This included a series of emails participants receive when they interacted with the communication system. Participants were prompted to explain the perceived goal of each component, what they noticed about the artifact, and what they would need to do next if they wanted to learn more or enroll in the study. During usability testing, we noted instances when participants did not know what to do next or when they reported an inaccurate take-away, informing modifications to system approachability.

To assess approachability for individuals with disabilities, we requested a review by an expert UX consultation service at the University of Washington. During the UX consultation, a team of UX experts assessed each component of the web-based communication system, with web accessibility in mind.

#### Acceptability

MAGENTA addressed the acceptability of genetic testing by enabling web-based participation over an internet-based communication system. By using a web-based communication system, the importance of provider identity diminished compared with in-person services, improving the acceptability of ovarian cancer genetic testing along the way. This has been the case for health services targeting a variety of stigmatized issues [25]. Web-based communication also might have made it easier to adapt and change messaging on an ongoing basis, modifying content to fit the needs of a diverse community. With this in mind, we opted to rotate through a diverse array of imagery and content when constructing web-based materials.

Web-based services invite an opportunity for branding. The MD Anderson MAGENTA website in particular served as a platform for building consumer trust and illustrating ties between the MAGENTA study and collaborating organizations, including familiar names such as MD Anderson and Stand Up To Cancer, sponsors of the study. Although potential participants may not have recognized MD Anderson, they may have heard of Stand Up To Cancer and may be more likely to consume and engage with information from a familiar source [26,27]. In favor of branding, we also chose to create a study specific Facebook page, lending credence to our social media presence. This page was linked to our paid Facebook advertising campaigns. Importantly, the study team also designed a logo for the MAGENTA study. This logo was used across all study materials to build familiarity and brand awareness.

Finally, we considered user privacy when designing the web-based communication system. Presumably, users prefer web-based tools with appropriate privacy protection. This was especially relevant when it came time to consider options for where to house data from study questionnaires and consent forms. With this goal in mind, we selected REDCap as the electronic data collection system [28]. REDCap is a Health Insurance Portability and Accountability Act–compliant platform, which is also compliant with Part 11 electronic signature regulations for the purposes of e-consent, features that made REDCap a natural fit for the MAGENTA study, addressing concerns about the management of confidential information.

All imagery and content were assessed through usability testing, with acceptability in mind. Participants reviewed images of the social media posts and advertisements. These materials were displayed on the computer screen and participants were asked to reflect on the images and content used and comment on what they noticed. Conversation was recorded alongside the screen recording, capturing what was being viewed. We also asked participants to identify collaborating institutions, or any familiar names associated with the study (eg, Stand Up To Cancer). We noted instances when participants made a positive or negative comment about the imagery or text used, as well as noting if they could identify the organizations behind the research effort. This information was used to inform modifications to images and content, changes tested in a second round of usability testing. Finally, we asked ovarian cancer advocates on the study team to review each web-based component. Insights from usability testing and advocates helped shape web-based materials.

#### Availability

We chose to use a web-based communication system to improve service availability [6]. Instead of requiring someone to have the means necessary to travel to a clinic for testing, the web-based communication system made it possible to learn about the study and participate in genetic testing via an internet connection. Participants could proceed through each web-based step at their own pace, without requirement to travel to any specific office. REDCap used automatic email reminders, making it easier to remind participants to complete various steps, simplifying longitudinal follow-up and potentially improving study attrition rates.

Similarly, the MAGENTA study team chose to offer genetic testing services through Color Genomics, a web-based genetic testing company, favoring the availability of web-based services over the more traditional clinic-based alternatives. Color Genomics is a company facilitating at-home genetic testing, along with web-based or phone-based counseling and education. Each step of this process, from ordering a saliva-based test kit to reviewing results, occurred on the web at the participant’s convenience. By giving participants the opportunity to complete genetic testing, including pre- and posttest genetic counseling via telehealth, MAGENTA effectively expanded the service range of genetic testing. Under this mode of provision, genetic testing was not just for those who live near a clinic offering this specialty service, it was available for anyone with an internet connection.

With availability in mind, we took precautions to ensure that each web-based component had the capacity to support high web traffic. Without this assurance, the MAGENTA study risked page crashes, reduced system availability, and, similarly, reduced availability of genetic testing services.

When dealing with a web-based system, availability extends beyond the physical availability of the service and into the usability of the system. In other words, a web-based communication system is only available to a user if it functions the way it is supposed to. To assess usability, we conducted usability testing of the web-based communication system. As part of the usability testing, participants were required to complete a web-based version of the Post-Study System Usability Questionnaire developed by Lewis [21]. This questionnaire provided quantitative data concerning system usability. We also referred the MD Anderson MAGENTA website and the electronic data collection system to expert UX consultation. The UX consultation focused on providing feedback on high-level workflows, page layout, and the usability of each web-based component. To encourage continued assessment of system usability, web analytics were tracked, on an ongoing basis across each web-based component in hopes of identifying and addressing any issues or bottlenecks before they impacted availability.

#### Affordability

For MAGENTA’s ovarian cancer genetic testing services to be widely affordable to women who may benefit, they must be available at no or low cost. Affordability is not just restricted to financial limitations but includes opportunity cost or the cost an individual is burdened with in exchange for accessing a service [29]. Additional barriers, such as cost of transportation, childcare, and work time lost, also play a role in the cost associated with the service, therefore reducing access. By offering genetic testing services on the web, we aim to reduce time lost and address secondary costs associated with in-person clinic visits, making genetic testing more affordable in terms of time and money. To ensure access, we also had to make sure that potential participants knew it was affordable. We tried to ensure that this information was included across study outreach efforts and other components of the web-based communication system, focusing on messaging such as “at no cost to you” and “genetic testing from your living room.”

Usability testing participants reviewed the web-based study components, focusing on outreach and background information (eg, social media posts or advertisements and study website), and were asked to reflect on the cost and location of research-related services, checking for comprehension and message clarity.

#### Appropriateness

MAGENTA staff created multiple images with diverse situations, appropriate for all social media outlets. This allowed us to rotate through a diverse array of imagery across each component, with the goal of creating an evolving narrative that resonated across different populations. We assessed imagery and content used throughout all social media advertisements, posts, and the study website for appropriateness through usability testing. During usability testing, we asked participants to talk through their initial reactions to imagery and posts, prompting them to discuss whether these components resonated with them, or to hypothesize who they might resonate for. That said, appropriateness invited ongoing assessment and modifications. Moving forward, the study team planned to use analytics to track the efficacy of different web-based components, informing modifications, and allowing the opportunity to tailor each component to ensure the overall system meets the needs of the targeted population.

### Ethics Approval

This research was reviewed by University of Washington Institutional Review Board, and determined to meet the criteria for exemption from the institutional review board review.

## Results

### Overview

The combined approach of usability testing and expert UX consultation resulted in a total of (1) 12 recommended changes to the social media advertisements and posts, (2) 34 recommended changes to the REDCap components of the system, and (3) 9 recommended changes across the MD Anderson MAGENTA website. Of these 55 recommendations, 36 (65%) addressed content (eg, imagery and text), 11 (20%) were related to web accessibility (eg, font and contrast), and 8 (15%) were related to page organization. All recommendations and design considerations were made with the goal of improving the overall accessibility of the web-based communication system, addressing potential barriers to the acceptability, availability, approachability, affordability, and appropriateness of the system, as defined by Levesque et al [13]. Table 2 summarizes these changes by accessibility component.

**Table 2:** Summary of accessibility concerns with current genetic testing practices and how Making Genetic Testing Accessible study addresses each concern.

Dimension	Barriers	Assessment	Decisions and adjustments
Approachability	Genetic testing is not approachable	Assess all advertisements and posts	Outreach routes should include social media, in-person (clinical settings and flyers), emails, and collaborations with trusted organizations (including ovarian cancer advocacy groups) Outreach materials should include physical flyers, paid social media advertisements, and unpaid posts across different social media channels
Acceptability	Genetic testing is not universally acceptable	Assess imagery and text used across all written study components	Use web-based system that (1) facilitates anonymity, (2) identifies study collaborators, (3) facilitates branding, (4) builds associations between study and related trusted organizations, (5) encourages responsive messaging, (6) facilitates rotating imagery and content, and (7) enables ongoing assessment Use an electronic data collection tool
Availability	Genetic testing is not universally available	Assess usability of each component and the whole system (buttons and links)	Use a web-based system that (1) meets accessibility standards (including font and device accessibility); (2) makes all web components physically available (eg, the Get Started button), (3) simplifies the email verification process, (4) enables reminders (including when to check email), and (5) includes a video explaining the study Use a web-based genetic testing service to make genetic testing itself physically available
Affordability	Genetic testing is not affordable to everyone	Assess content related to cost of services	Genetic testing is free to participants
Appropriateness	Genetic testing is not appropriate for everyone	Assess imagery and text used across all components	Use a diverse array of imagery (including families, uplifting, women aged >30 years, and candid images) Avoid fear-based language Make modifications to outreach materials based on performance

### Approachability

Usability testing participants were unclear about what service was being offered when just viewing the advertisements. A participant noted, “I don’t know if this is about research or they’re trying to sell me something,” and another said, “what is Facebook doing thinking about my ovaries?” With these comments in mind, we updated the advertisement content to reflect the research goals more appropriately. We monitored responses to the advertisement and adjusted as needed.

Participants also noted where the font was difficult to read or decode, issues that were also identified through our expert UX consultation, which provided substantial insight regarding web accessibility changes. Many of the web accessibility recommendations were not able to be supported in REDCap. Although we could update the font color over REDCap, other changes, such as removing the font resize function built into REDCap surveys, were not possible. Although REDCap supported the confidential collection of data, enhancing the acceptability of the system, our assessments indicated that REDCap has room for improvement in terms of system approachability, particularly regarding web accessibility.

### Acceptability

In terms of acceptability, several usability testing participants mentioned that they did not know who *MD Anderson Cancer Center* or *Stand Up To Cancer* was. Others were unsure what MAGENTA was when seeing this name connected to Facebook and social media outreach. Many usability testing participants commented that they felt unsure about who was behind the study and felt uneasy about relinquishing their genetic data to a web-based entity. This observation led to some content revisions, with the goal of highlighting the organizations behind the research more clearly on the MD Anderson MAGENTA website. We tracked traffic across each component of the multistep web-based communication system, via web analytics. This enabled us to identify which components are working well and where there might be problems.

### Availability

In general, participants reported that the progression from social media posts, or advertisements, to the MD Anderson website was easy to understand. Although the progression was clear, participants still made some recommendations addressing system availability. For example, several participants noted that it was difficult to figure out what they needed to do next on the MD Anderson MAGENTA website and suggested that the *Do I qualify?* button be relabeled and moved to a more prominent position. This suggestion was also brought up during our expert UX consultation. We addressed this problem by relabeling the button *Get Started* and by moving the button to a more central location that was easier to find.

Expert user consultation combined with usability testing also led to several recommendations that addressed the usability and flow of the electronic data collection system. System usability was also captured via the Post-Study System Usability Questionnaire developed by Lewis [21]. Participants who completed the questionnaire (N=7) indicated that they were moderately satisfied with the system ease of use and efficiency (5.06, where 1 indicates low satisfaction and 7 indicates high satisfaction). When asked about information quality, meaning they had the information they needed to complete the task or interact with the system, participants reported moderate satisfaction again (4.88). Participants reported lower levels of satisfaction when asked about the quality of the interface (4.56). Overall, participants were moderately satisfied with the system, with a mean of 4.91 (SD 0.35) across all items.

### Affordability

When assessing the affordability of the system, usability testing participants could accurately identify that the services rendered through study participation were free of charge and available to complete on their own time and at their own pace. To demonstrate this, participants commented on genetic testing being available at “no cost.”

### Appropriateness

When assessing the appropriateness of the system, usability test participants noted that images with a more natural appearance, as opposed to those that were clearly posed, were easier to relate to. When speaking about a more candid image, a participant said, “I like that image, because it looks more realistic.” Another participant commented on the age of a mother-child dyad featured in an image, stating, “I’m unclear why there is a mom and a baby, when this probably should be for women with teenagers.” When looking at an image featuring a mother with a young baby, another participant pointed out that a mother-infant dyad such as this might exclude women without children, who may still be at risk of ovarian cancer. Most participants who noted the diversity across images remarked positively about the representation, with one noting, “I like that I see a lot of diversity in these images.”

Usability test participants and ovarian cancer advocates spoke favorably about content that framed ovarian cancer as something that is relevant to anyone with a family history. They felt that using a picture of a family in this context suggested that the actions of one individual could benefit others in their family. Insight gained from this discussion led to the decision to focus on images that featured >1 person or families, as well as images that appeared to be more candid. The ovarian cancer advocates also noted that the *fear*-based language present in earlier iterations of the paid advertisements and unpaid posts, such as “Are you at risk for ovarian cancer?”, would be less likely to inspire them into action, when compared with something more positive or hopeful, such as a message imploring people to work as a team toward a solution. This note was echoed among ovarian cancer advocates that we consulted with and informed changes to content.

## Discussion

### Principal Findings

The accessibility of genetic testing continues to be a challenge, leaving many eligible candidates faced with barriers to participation. Current research suggests that the internet may address many of the components of accessibility outlined by Levesque et al [13]. With a paucity of research dedicated to assessing the efficacy of multistep web-based communication systems for facilitating genetic testing, careful planning and consideration were required. The recommendations generated through this assessment focused primarily on the organization and usability of the system, and there was continued assessment over the course of recruitment. The success of the multistep web-based communication system could help facilitate genetic testing implementation in the future, increasing the use of the internet for physician-mediated genetic counseling and genetic testing, ultimately leading to increased access to genetic testing, particularly among populations currently underserved by genetic testing.

Applications of telehealth, such as those evaluated through this research, address critical barriers and improve accessibility [30]. Telehealth has become even more important during the COVID-19 pandemic, with the halt of in-person activities in research and health care settings, in the interest of disease mitigation [30]. The web-based communication system evaluated for use in the MAGENTA study was fully remote, leveraging some of the positive attributes of telehealth services. Although the usability testing described in this paper was conducted in person, the described methods can be implemented remotely, improving access among adults with an internet connection and providing an opportunity for a more diverse subject population. This accommodation does not address access among those who do not have access to the internet, and additional research is needed to identify innovative approaches for reaching these individuals.

### Limitations

We assessed the strengths and weaknesses of the evaluation process along the way. Although we attempted to address issues with the evaluation process as they emerged, usability testing had its limitations. For parts of usability testing, we had to rely on screenshots of systems or use systems that were not functional. Instead of proceeding through the multistep system in these situations, we had participants talk about what they expected or what they would interact with if the system was active. Thus, we were unable to determine how long it took users to complete certain tasks, both in time elapsed and number of clicks. This was one reason the study team, in partnership with patient advocates, remain dedicated to the ongoing assessment of the multistep web-based communication system. By continuing the assessment and inviting modification as needed, focusing on web analytics to identify bottlenecks and attrition, we aim to address any issues that usability testing failed to identify, which may arise through further system use and increased traffic to different components of the web-based communication system.

Participants spoke favorably of the use of diverse imagery, featuring Black and indigenous people of color. Diverse imagery is known to be an important feature to ensure a message resonates with a wider research participant population. Although MAGENTA outreach materials ultimately featured people from different racial-ethnic backgrounds, the research recruited a predominantly White study participant population [31]. With this observation in mind, it is clear that diverse imagery alone cannot address the widespread and complex issue of underrepresentation in research. Racial and ethnic minorities are often underrepresented in genetic testing studies and services [32,33].

The eligibility criteria implemented in usability testing contributed to another limitation related to representation. The MAGENTA study specifically recruited women with at least one ovary, effectively excluding transgender men who may also be at an increased risk of ovarian cancer. The decision to exclude transgender men from participating in the MAGENTA study was made in response to the different health needs exhibited between cisgender women and transgender men. In addition, the standardized instruments used in the larger MAGENTA study were not validated across transgender and nonbinary populations. As the eligibility criteria for usability testing loosely mirrored the larger study, we made the decision to specifically include cisgender women. The exclusion of transgender and nonbinary individuals from reproductive cancer research, and subsequent health care services is a significant limitation that contributes to adverse health outcomes for these individuals, perpetuates health disparities, and requires attention.

Similarly, usability testing did not recruit participants based on health insurance status, reflecting the eligibility criteria in the MAGENTA study. MAGENTA did not formally require participants to have insurance; however, all research participants were required to provide the name and contact information of a health care provider. This step may have functionally excluded uninsured individuals. Although the MAGENTA study addressed the issue of affordability to a certain extent by providing genetic testing to participants at no cost, usability testing arguably did not fully evaluate this dimension of accessibility, as financial barriers do not stop with the cost of testing but also encompass the cost associated with increased cancer surveillance that a positive genetic test result may catalyze. Further assessment is needed to evaluate web-based communication platforms, with these underrepresented populations in mind.

Usability testing is poised to help address this trend, given the focus on identifying communication and system barriers. To effectively address barriers for underrepresented racial and ethnic groups, usability testing needs to be conducted with a representative population that includes racial and ethnic minorities. Given the predominantly White population enrolled in this usability study and the parallel research participant population enrolled through the MAGENTA study, it appears that the approachability and appropriateness dimensions in this usability study were limited. Future usability testing for web-based genetic testing research should focus on assessing barriers that are specific to underrepresented groups, including underinsured and uninsured individuals, as well as Black and indigenous people of color, in the interest of better assessing the accessibility barriers.

## Abbreviations

MAGENTA: Making Genetic Testing Accessible
REDCap: Research Electronic Data Capture
UX: user experience
